# Relationship of Regular Laxative Use, Genetic Susceptibility of Depression, and Risk of Incident Depression in the General Population

**DOI:** 10.1155/2024/6863037

**Published:** 2024-10-23

**Authors:** Yuanyuan Zhang, Xiaoqin Gan, Chun Zhou, Ziliang Ye, Panpan He, Mengyi Liu, Yanjun Zhang, Sisi Yang, Xianhui Qin

**Affiliations:** Division of Nephrology, Nanfang Hospital, Southern Medical University, National Clinical Research Center for Kidney Disease, State Key Laboratory of Organ Failure Research, Guangdong Provincial Institute of Nephrology, Guangdong Provincial Key Laboratory of Renal Failure Research, Guangzhou 510515, China

**Keywords:** depression, genetic susceptibility, microbiome–gut–brain axis, regular laxative use, sex

## Abstract

**Background:** The relationship between laxative use and the risk of depression remains uncertain. We aimed to assess the prospective association of regular laxative use with the risk of incident depression and to examine whether genetic risk of depression modifies this association.

**Methods:** Four hundred fifty thousand forty-five participants without depression at baseline and have complete information on laxative use from the UK Biobank were included. The study outcome was incident depression, derived from linkage to primary care records, hospital inpatient data, death register records, or self-reported medical conditions at follow-up visits.

**Results:** During a median follow-up of 12.4 years, 18,651(4.1%) participants have developed depression. Regular laxative use was significantly associated with a higher risk of incident depression (vs. nonregular laxative use; adjusted hazard ratio [HR] = 1.78, 95% confidence interval [CI], 1.68–1.89). Genetic risk of depression did not significantly modify this association. The risk of incident depression increased with increasing types of laxatives used, with a HR of 1.89 (95%CI, 1.73–2.08) for use of single laxative type and 2.32 (95%CI, 1.82–2.96) for combined use of two or more laxative types (*P* for trend <0.001). The positive association between regular laxative use and incident depression was more pronounced in men (adjusted HR = 2.21, 95%CI, 1.96–2.48) versus women (adjusted HR = 1.67, 95%CI, 1.56–1.79; *P* interaction <0.001). Compared to those who did not use laxatives regularly and did not have constipation, participants who used laxatives regularly and had constipation had the highest risk of incident depression (adjusted HR = 2.33, 95%CI, 1.94–2.80).

**Conclusions:** Regular laxative use was significantly associated with a higher risk of incident depression, especially in men.

## 1. Introduction

Depression is a substantial global health concern that severely limits psychosocial functioning, reduces quality of life, and is a leading cause of disability and premature mortality worldwide [1–3]. It is reported that about 280 million people suffered from depression in 2019, and the prevalence of depression is still increasing [4, 5]. Given the variety of manifestations of depression, its unpredictable course and prognosis, and the limited effectiveness of treatment, primary prevention of depression based on the identification of more modifiable factors is critical.

In recent years, the association between gut microbiota or bowel dysfunctions and mental health has received increasing attention. Accordingly, irritable bowel syndrome (IBS) [6], inflammatory bowel disease (IBD) [7], and constipation [8] have been reported to be associated with depression in several previous studies. Interestingly, the use of laxatives can lead to a new steady state of gut microbiota composition and long-term changes in the adaptive immune response [9]. Recent studies have also shown that alterations in gut microbiota can affect brain and mental health through microbial regulation of neuroimmune signaling, microbiota-mediated tryptophan metabolism, and microbial-controlled neuroendocrine function [10]. These multiple pathways on the microbiome–gut–brain axis [11] support the hypothesis that regular laxative use may be associated with the risk of depression. However, to date, few studies have explored the relationship of regular laxative use with the risk of depression in the general population. In addition, depression is a complex, multifactorial disease resulting from the interaction of genetic and environmental factors [12]. Whether genetic susceptibility of depression modifies the association between regular laxative use and depression risk remains unknown.

Our current study was motivated by limited data on regular laxative use and incident depression and an exceptional opportunity to address this question in a large, population-based cohort with nearly half a million populations. Specifically, using data from the UK Biobank, we sought to evaluate the association between regular laxative use and the risk of incident depression in the general population and to explore the possible effect modifiers on this association.

## 2. Methods

### 2.1. Study Design and Participants

Our current study was derived from the UK Biobank, a prospective, nationwide cohort of approximately half a million participants aged 37–73 years from 22 study centers across England, Wales, and Scotland between 2006 and 2010. At baseline, all participants were invited to complete a touchscreen questionnaire, a face-to-face nurse interview, and a series of physical measurements and to provide biological samples for laboratory measurements. The details of the study design and data collection have been described in the official website (https://www.ukbiobank.ac.uk/) and previous studies [13–17]. The UK Biobank was approved by the North West Multicenter Research Ethics Committee (11/NW/0382), and all participants were informed at the initial of the study and signed an informed consent.

Of the 502,461 participants enrolled in the UK Biobank, those who withdraw from the study (*n* = 107) and who had depression at baseline (*n* = 42,991) were excluded. Furthermore, 9318 participants who had no information on use of laxatives were further excluded. Therefore, 450,045 participants were included in the final analyses (Figure S1).

### 2.2. Assessments of Regular Laxative Use

The information on the regular use of laxatives was collected in the touchscreen questionnaire and face-to-face nurse interview at baseline. For data on the touchscreen questionnaire, under the section of “medications for pain relief, constipation, heartburn,” each participant was asked, “Do you regularly take any of the following?”, and then they could select more than one answer from a list of medications (including aspirin, laxatives, and ibuprofen). In the questionnaire, regular use means use on most days of the week for the last 4 weeks. Regular laxative use was defined as 1 = true and 0 = false.

For data on face-to-face nurse interview, if a participant reported any regular medication intake in the touchscreen, the names of the medication were recorded. If the participant stated in the touchscreen questionnaire that they were not taking any regular prescription medications (or were not sure), this question was asked again and confirmed by the interviewer. Based on the names of the medication reported by the participant, according to the British National Formulary (BNF) [18], we collected four common subtypes of regular used laxatives, including fecal softeners, bulk-forming laxatives, osmotic laxatives, and stimulant laxatives [19].

### 2.3. Ascertainment of Depression

The study outcome was incident depression, ascertained by self-reported medical conditions at follow-up visits and date linkage to primary care records, hospital inpatient data, and death register records. Of which, hospital inpatient data were based on the International Classification of Diseases edition 10 (ICD-10), coded as F32 or F33. More details about the ascertainment of the outcome can be found online (https://biobank.ndph.ox.ac.uk/showcase/label.cgi?id=2405).

The follow-up for each participant was calculated from the date of first assessment until the first date of incident depression, date of death, date of lose to follow-up, or the end of follow-up, whichever came first.

### 2.4. Genetic Predisposition of Depression

Genetic risk scores (GRSs) for depression were created using a weighted method [20], by 37 single-nucleotide polymorphisms (SNPs) that showed independently significant genome-wide association with depression [21, 22]. A higher GRS indicated a higher genetic predisposition to depression. Participants were divided into high, moderate, or low genetic risk for depression according to the tertiles of depression GRS. Detailed information about genotyping and quality control in the UK Biobank study has been described previously [23].

### 2.5. Measurements of Covariates

Data on age, sex, race, height, weight, education levels, household incomes, Townsend deprivation index (TDI), smoking status, and alcohol consumption, serum C-reactive protein (CRP), lipids, physical activity, healthy diet scores, history of abdominal operation, and concomitant diseases (diabetes, hypertension, cardiovascular disease [CVD], dementia, constipation, and IBD) were available through standardized questionnaires, physical measurements, and measures of biological samples.

Body mass index (BMI) (kg/m^2^) was calculated by dividing weight (kg) by square of standing height (m). Hypertension at baseline was defined as systolic blood pressure (SBP) ≥140 mmHg, or diastolic blood pressure (DBP) ≥90 mmHg, or self-reported antihypertensive treatment, or self-reported hypertension diagnosis, or a diagnosis based on ICD-9 (401) or ICD-10 (I10). Diabetes was defined as prevalent diabetes [24] or hemoglobin A1c (HbA1c) ≥6.5%. CVD at baseline was identified as a composite of pre-existing coronary heart disease, myocardial infarction, stroke, and heart failure. Constipation was defined as self-reported constipation diagnosis or a diagnosis based on ICD-9 (5640) or ICD-10 (590). The healthy diet score was calculated by considering adequate consumption of fruits, vegetables, whole grains, fish, shellfish, dairy products, and vegetable oils and reduced consumption of refined grains, processed meats, unprocessed meats, and sugar sweetened beverages [25]. If participants met one of 10 dietary goals, they will get one point, with a maximal of 10 points.

Procedures for collecting and processing blood and urine samples have been previously described and validated [26]. All blood samples were measured at a dedicated central laboratory. Serum CRP was measured by immunoturbidimetric method, lipids were measured by cholesterol oxidase–peroxidase method analysis, and HbA1c was measured by the high-performance liquid chromatography method. More details about these covariates can be found in the UK Biobank online protocol (https://www.ukbiobank.ac.uk).

### 2.6. Statistical Analysis

#### 2.6.1. Main Analyses

Baseline characteristics of study participants were shown as mean ± standard deviations (SDs) for continuous variables and number (proportions) for categorical variables. Differences in population characteristics by regular laxative use (yes vs. no) were compared using *t*-test for continuous variables and chi-square tests for categorical variables, respectively.

The hazard ratios (HRs) and 95% confidence intervals (CIs) of incident depression with regular use of any laxatives and four common subtypes of laxatives (bulk-forming laxatives, fecal softeners, osmotic laxatives, and stimulant laxatives) were estimated using Cox proportional hazards models. Variables known to be traditional or suspected risk factors for depression were selected as covariates in the analysis. Model 1 included the adjustments for age, sex, and race. Model 2 included the adjustments for age, sex, race, BMI, education levels, household incomes, TDI, smoking and alcohol consumption status, family history of depression, physical activity, healthy diet scores, low-density lipoprotein cholesterol (LDL-C), triglyceride, and CRP, as well as the prevalence of hypertension, diabetes, CVD, dementia, and IBD. Stratified analyses and interactions test were applied to explore the potential effect modifiers on the association of regular laxative use and incident depression.

#### 2.6.2. Sensitivity Analyses

To test the robustness of our findings, a series of sensitivity analyses were conducted. First, since the regular use of laxatives is a proxy of chronic constipation, the link between laxatives and depression, if it exists, may be modified by constipation status. To examine this possibility, we made a stratified analysis by constipation status and explored the joint effect of regular laxative use and constipation status on incident depression. Second, participants who were followed for 2 years or less or 5 years or less were excluded to minimize reverse causality. Third, the depression GRS or history of abdominal operation was further adjusted. Fourth, propensity score matching method was performed to further confirm the results. A nonparsimonious propensity score was developed using variables that might affect laxative use or incident depression, including age, sex, race, BMI, education levels, household incomes, TDI, smoking, alcohol consumption, family history of depression, physical activity, healthy diet scores, LDL-C, triglyceride, and CRP, as well as the prevalence of hypertension, diabetes, CVD, IBD, and dementia, to predict the likelihood that a participant would be in the different status (use or nonuse) of laxative use, respectively. Participants were matched 1:1 based on propensity scores. An automated balance optimization method using the function Match (in package MatchIt) in R and a caliper of 0.2 was used for matching. Standardized differences of postmatched participant characteristics ≤10% between the two groups were considered to be balanced. In addition, we also performed stratified analyses and interaction testing to explore possible modifications on the association between regular laxative use and risk of incident depression.

A two-tailed *p* < 0.05 was considered to be statistically significant in all analyses. Analyses were performed using R software (version 4.1.1, http://www.R-project.org/).

## 3. Results

### 3.1. Study Participants and Baseline Characteristics

Of the 450,045 participants included in the current study (Figure S1), the mean age was 56.6 (SD, 8.1) years, 209,411 (46.5) were male, and 15,205 (3.4%) participants reported regular use of any laxatives at baseline.

Regular laxative users were older, more likely to be female and current smokers, and had higher BMI, TDI, and CRP levels; higher frequency of family history of depression; higher prevalence of hypertension, diabetes, CVD, and IBD; and lower physical activity, income and education levels, and lower alcohol consumption (Table 1).

### 3.2. Association Between Regular Use of Laxatives and Incident Depression

During a median follow-up of 12.4 (interquartile range, 11.6–13.2) years, 18,651(4.1%) participants occurred depression. Regular laxative use (v. nonregular laxative use; adjusted HR = 1.78, 95%CI, 1.68–1.89) was significantly associated with a higher risk of incident depression (Table 2).

Of the 15,205 regular laxative users, 6045 participants reported that they were taking specific types of laxatives. Of these, 5412 participants used only one type of laxative, and 633 used two or more type of laxatives. The risk of incident depression increased with increasing types of laxatives used, with a HR of 1.89 (95%CI, 1.73–2.08) for use of single laxative type and 2.32 (95%CI, 1.82–2.96) for combined use of two or more laxative types (*P* for trend <0.001, Table 2).

In addition, in a comparison of regular use of each laxative type versus nonregular use of laxatives, regular use of each of the four common laxative subtypes, including bulk-forming laxatives (adjusted HR = 1.82, 95%CI, 1.56–2.13), fecal softeners (adjusted HR = 2.08, 95%CI, 1.43–3.04), osmotic laxatives (adjusted HR = 2.04, 95%CI, 1.75–2.37), and stimulant laxatives (adjusted HR = 1.75, 95%CI, 1.43–2.13), was significantly associated with a higher risk of incident depression (Table 3).

#### 3.2.1. Sensitivity Analysis

In the propensity score analysis, 27,806 participants (13,903 in each group) were included in the analysis of the association between regular use of laxatives (vs. nonregular use) and incident depression. Candidate variables used in the development of the propensity score have been listed in Table S1. All the postmatched participant characteristics were highly balanced (Figure S2). Consistently, a significantly higher risk of incident depression was found in participants with regular use of laxatives (8.7% vs. nonregular use 5.0%; adjusted HR = 1.79, 95%CI, 1.63–1.96) (Sensitive analysis 1 in Table S2).

Excluding participants who were followed for 2 years or less (Sensitive analysis 2 in Table S2), or 5 years or less (Sensitive analysis 3 in Table S2), or excluding those who had constipation at baseline or during follow-up (Sensitive analysis 4 in Table S2), or further adjusting for depression GRS (Sensitive analysis 5 in Table S2), or history of abdominal operation (Sensitive analysis 6 in Table S2) did not materially change the findings.

### 3.3. Joint Effect of Regular Laxative Use and Constipation Status on the Risk of Incident Depression

**Figure 1:**
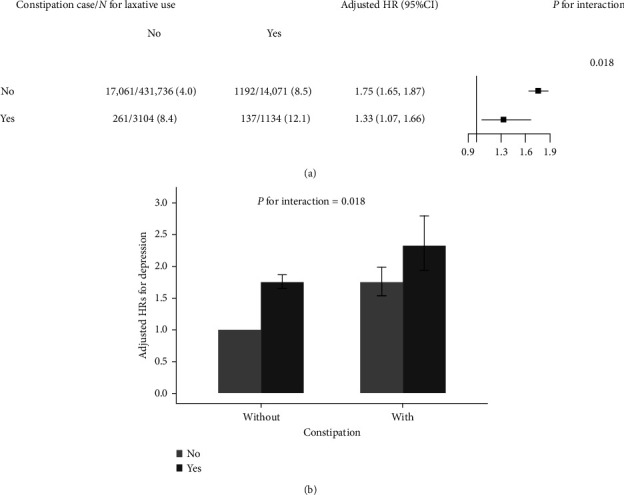
Adjusted for age, sex, race, body mass index, education levels, household incomes, Townsend deprivation index, smoking and alcohol consumption status, family history of depression, physical activity, healthy diet scores, levels of low-density lipoprotein cholesterol, triglyceride and C-reactive protein, prevalence of hypertension, diabetes, cardiovascular disease, inflammatory bowel disease, and dementia. (A) Stratified analysis by constipation status on the relationship of regular laxative use and the risk of incident depression and (B) Joint effect of regular laxative use and constipation status on the risk of incident depression. HR, hazard ratio.

A stronger positive association between regular laxative use and incident depression was found in participants without constipation at baseline (adjusted HR = 1.75, 95%CI, 1.65–1.87, vs. those with constipation, adjusted HR = 1.33, 95%CI, 1.07–1.66; *P* for interaction = 0.018, Figure 1A). Accordingly, compared to those who did not use laxatives regularly and did not have constipation, participants who used laxatives regularly and had constipation had the highest risk of incident depression (adjusted HR = 2.33, 95%CI, 1.94–2.80, Figure 1B).

### 3.4. Stratified Analyses of the Association Between Regular Laxative Use and Incident Depression

**Figure 2:**
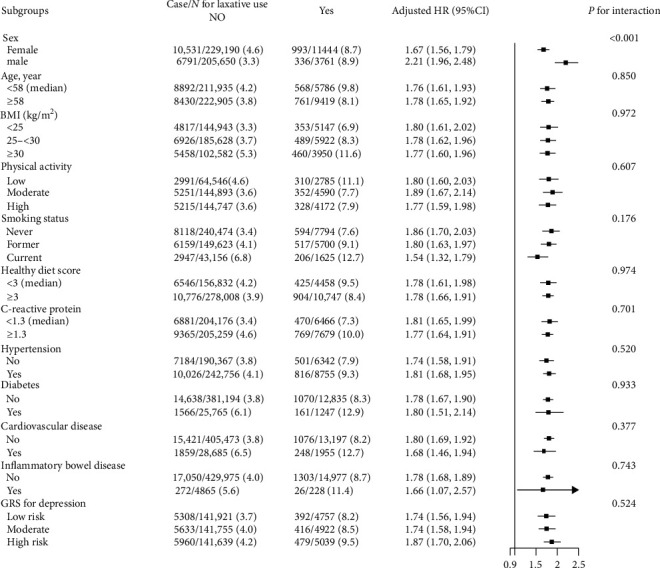
Stratified analyses of the association between regular laxative use and the risk of incident depression. Adjusted, if not stratified, for age, sex, race, body mass index, education levels, household incomes, Townsend deprivation index, smoking and alcohol consumption status, family history of depression, physical activity, healthy diet scores, levels of low-density lipoprotein cholesterol, triglyceride and C-reactive protein, prevalence of hypertension, diabetes, cardiovascular disease, inflammatory bowel disease, and dementia. BMI, body mass index; GRSs, genetic risk scores; HR, hazard ratio.

The positive association between regular laxative use and incident depression was more pronounced in males (adjusted HR = 2.21, 95%CI, 1.96–2.48, vs. female, adjusted HR = 1.67, 95%CI, 1.56–1.79; *P* for interaction <0.001) (Figure 2).

None of the other variables, including age (<58 vs. ≥58 years, median), BMI (<25 vs. 25–<30 vs. ≥30 kg/m^2^), physical activity (low vs. moderate vs. high), smoke status (never vs. former vs. current), healthy diet score (<3 vs. ≥3 mg/L, median), CRP concentrations (<1.3 vs. ≥1.3 mg/L, median), hypertension (no vs. yes), diabetes (no vs. yes), CVD (no vs. yes), and IBD (no vs. yes) at baseline and genetic risk of depression (low vs. moderate vs. high risk), significantly modified the association between regular use of laxatives and the risk of incident depression (all *P* interactions >0.05) (Figure 2).

## 4. Discussion

Findings from a large sample, long follow time study in UK Biobank showed that regular laxative use, including any subtype of laxatives, was significantly associated with a higher risk of incident depression, and the risk increased with the number of laxative types used. Furthermore, there was a stronger positive association between regular laxative use and incident depression in men.

A cross-sectional survey from the National Health and Nutrition Examination Survey (NHANES) of 6765 participants who were attempting to lose weight reported that unhealthy weight loss strategies, including the use of laxatives, were associated with increased odds of depression [27]. However, to date, few studies have examined the prospective association between laxative use and the risk of incident depression in the general population. Our current study, which took into account regular use of any laxative and four common laxative subtypes, showed that regular use of laxatives, including any type of laxative, was associated with a higher risk of depression.

Since regular laxative use suggests the possibility of chronic constipation, we speculate that the observed association between laxative use and depression may be modified by constipation status. We found that the positive association of laxative use with depression was more pronounced in participants without constipation. Since all laxatives are over-the-counter, laxative abuse is common among middle-aged and older adults. The reason for the use of laxatives by participants who were not constipated may be that these individuals were more convinced that daily defecation was necessary for physical health [28]. Although the association of laxative use with incident depression was slightly weaker in participants with constipation, participants who regularly used laxatives and had constipation had the highest risk of incident depression. Moreover, prior studies have reported that constipation was associated with a higher risk of depression [29, 30]. Therefore, we inferred the detrimental effects of laxative on depression were partly masked by constipation. Taken together, these findings suggest that the observed association between laxatives and depression is more likely to reflect the potential effects of laxatives per se rather than just the relationship of constipation with depression.

The underlying mechanisms by which laxative use increases the risk of depression is biologically plausible. Laxatives may affect the composition of the gut microbiome [9, 31] and therefore affect the brain and mental through the microbiome–gut–brain axis health [10, 11]. Moreover, changes in the composition of gut microbiota may lead to changes in the permeability of the intestinal barrier, resulting in an imbalance in the amount of gut peptides [32]. Gut peptides, such as peptide YY (PYY), glucagon-like peptide (GLP-1), cholecystokinin (CCK), corticotropin-releasing factor (CRF), ghrelin, and oxytocin, had been shown to regulate endocrine activity and communicate with the central nervous system [32, 33]. For example, depression is associated with hyperactivity of CRF neuronal pathways [34]. In addition, gut dysbiosis affects the modulation of nerve signaling and the production of numerous neurotransmitters (such as gamma-aminobutyric acid, serotonin, dopamine, tryptophan metabolites, catecholamines), which may penetrate into the bloodstream and act directly on receptors in the brain [35]. More studies are needed to confirm our findings and further investigate the underlying mechanisms.

More interestingly, the positive association between laxative use and risk of incident depression was more pronounced in men. Since women have a higher absolute risk of depression, we speculate that the harmful effects of laxative use may be partially masked in women, a population at high risk for depression [36]. However, it should be seen that laxative use was still significantly associated with a high risk of depression even in women. Therefore, laxative use should be closely monitored regardless of sex to prevent its associated increased risk of depression.

Several limitations need to be addressed. First, given the nature of observational studies, although a range of possible confounders were adjusted in the analysis and propensity score matching method was used, residual confounding of unknown or unmeasured factors cannot be exclude. Second, data on the dosage of laxative use were not available, and we therefore could not test the dose–response relationship of the use of laxatives with depression risk. Moreover, the regular use of laxatives was from questionnaires. And this questionnaire is not purposively designed to get the information of regular use of laxatives. Thus, information bias might exist. Third, the participants were predominantly Caucasian, which limits the generalizability of the results to other geographic regions or ethnicities. Fourth, in our study, the assessment of depression was based on self-report medication use, primary care records, and ICD codes, which might have contributed to some undetected depressive events. This potential for misclassification of patients with depression may attenuate findings toward the null, leading to an underestimation of magnitudes of the true association. Finally, as not all those who reported regular use of laxatives can be tracked back to get the information of the specific types of laxatives, conclusions about the relationship between the types of laxatives and more than two types of laxatives with the risk of depression need to be further confirmed in future studies. In addition, more studies are needed to clarify whether the association between laxative use and depression risk is causal.

In conclusion, regular laxative use was associated with a higher risk of incident depression, especially among men. Our findings highlight the importance of monitoring mental health while using laxatives and have important clinical value for primary prevention of depression.

## Figures and Tables

**Table 1:** Baseline characteristics of the study participants.

Baseline characteristics*⁣*^*∗*^	Regular laxative use, *n* (%)		*P* values
	No	Yes	
*N*	434,840	15,205	—
Age, years	56.6 ± 8.1	58.7 ± 7.7	<0.001
Male,*n* (%)	205,650 (47.3)	3761 (24.7)	<0.001
Body mass index (kg/m^2^)	27.3 ± 4.7	27.6 ± 5.2	<0.001
White, *n* (%)	410,254 (94.3)	14,050 (92.4)	<0.001
Townsend deprivation index	−1.4 ± 3.0	−0.9 ± 3.3	<0.001
College or university degree, *n* (%)	143,864 (33.1)	3528 (23.2)	<0.001
Income less than 18,000£, *n* (%)	78,936 (18.2)	4250 (28.0)	<0.001
Healthy diet score	3.0 ± 1.4	3.2 ± 1.3	<0.001
Family history of depression, *n* (%)	50,373 (11.6)	2303 (15.1)	—
Smoke status, *n* (%)	—	—	<0.001
Never	240,474 (55.3)	7794 (51.3)	—
Ever	149,623 (34.4)	5700 (37.5)	—
Current	43,156 (9.9)	1625 (10.7)	—
Alcohol drinking, *n* (%)	—	—	<0.001
Never	32,316 (7.4)	1961 (12.9)	—
<1 time per week	95,417 (21.9)	4423 (29.1)	—
1–4 times per week	216,753 (49.8)	6369 (41.9)	—
Daily or almost daily	90.031 (20.7)	2423 (15.9)	—
Physical activity, *n* (%)	—	—	<0.001
Low	64,546 (18.2)	2785 (24.1)	—
Moderate	144,893 (40.9)	4590 (39.8)	—
High	144,747 (40.9)	4172 (36.1)	—
Laboratory results	—	—	—
C-reactive protein (mg/L)	2.5 ± 4.3	3.0 ± 4.9	<0.001
Low-density lipoprotein cholesterol (mmol/L)	3.6 ± 0.9	3.5 ± 0.9	<0.001
Triglyceride (mmol/L)	1.7 ± 1.0	1.7 ± 1.0	<0.001
History of disease, *n* (%)	—	—	—
Dementia	161 (0.0)	16 (0.1)	—
Hypertension	242,756 (56.0)	8755 (58.0)	<0.001
Diabetes	25,765 (6.3)	1247 (8.9)	<0.001
Cardiovascular disease	28,685 (6.6)	1955 (12.9)	<0.001
Inflammatory bowel disease	4865 (1.1)	228 (1.5)	<0.001
Depression genetic risks	—	—	0.015
Low	141,921 (33.4)	4757 (32.3)	—
Moderate	141,755 (33.3)	4922 (33.4)	—
High	141,639 (33.3)	5039 (34.2)	—

*⁣* ^*∗*^Continuous variables are presented as mean ± standard deviations (SDs); category variables are presented as *n* (%).

**Table 2:** Relationship of regular laxative use and number of laxative types used with the risk of incident depression.

Regular laxative use	*N*	Events (%)	Model 1		Model 2	
			HR (95%CI)	*P* value	HR (95%CI)	*P* value
Regular laxative use						
No	434,840	17,322 (4.0)	Ref	—	Ref	—
Yes	15,205	1329 (8.7)	2.18 (2.06, 2.31)	<0.001	1.78 (1.68, 1.89)	<0.001
Number of laxative types						
No regular use	434,840	17,322 (4.0)	Ref	—	Ref	—
Single type	5412	504 (9.3)	2.39 (2.18, 2.61)	<0.001	1.89 (1.73, 2.08)	<0.001
Two or more types	633	82 (13.0)	3.56 (2.87, 4.43)	<0.001	2.32 (1.82, 2.96)	<0.001
Unspecified	9160	743 (8.1)	1.98 (1.84, 2.14)	<0.001	1.68 (1.55, 1.81)	<0.001
*P* for trend	—	—	<0.001	—	<0.001	—

*Note*: Model 1: adjusted for age, sex, and race. Model 2: adjusted for age, sex, race, body mass index, education levels, household incomes, Townsend deprivation index, smoking and alcohol consumption status, family history of depression, physical activity, healthy diet scores, levels of low-density lipoprotein cholesterol, triglyceride and C-reactive protein, prevalence of hypertension, diabetes, cardiovascular disease, inflammatory bowel disease, and dementia.

Abbreviation: HR, hazard ratio.

**Table 3:** Association between regular use of specific types of laxatives and the risk of incident depression*⁣*^*∗*^.

Laxative types	*N*	Events (%)	Model 1		Model 2	
			HR (95%CI)	*P* value	HR (95%CI)	*P* value
No regular use	434,840	17,322 (4.0)	Ref	—	Ref	—
Bulk-forming	2139	178 (8.3)	2.07 (1.78, 2.39)	<0.001	1.82 (1.56, 2.13)	<0.001
Fecal softeners	285	31 (10.9)	3.00 (2.11, 4.26)	<0.001	2.08 (1.43, 3.04)	<0.001
Osmotic	1837	183 (10.0)	2.59 (2.24, 3.00)	<0.001	2.04 (1.75, 2.37)	<0.001
Stimulant	1151	112 (9.7)	2.51 (2.08, 3.02)	<0.001	1.75 (1.43, 2.13)	<0.001

*Note*: Model 1: adjusted for age, sex, and race. Model 2: adjusted for age, sex, race, body mass index, education levels, household incomes, Townsend deprivation index, smoking and alcohol consumption status, family history of depression, physical activity, healthy diet scores, levels of low-density lipoprotein cholesterol, triglyceride and C-reactive protein, prevalence of hypertension, diabetes, cardiovascular disease, inflammatory bowel disease, and dementia.

*⁣* ^*∗*^This analysis included 434,840 participants with no regular use of laxatives and 5412 participants who reported the use of single type of laxatives.

## Data Availability

The UK Biobank data are available on application to the UK Biobank (https://www.ukbiobank.ac.uk/), and the analytic methods and study materials that support the findings of this study will be available from the corresponding authors on request.

## Supporting Information

Supporting Information Figure S1. Flow chart of the study participants. Figure S2. Standardized differences of participant characteristics before and after matched according to propensity scores by laxative use. Table S1. Postmatched participant characteristics according to propensity scores by laxative use. Table S2. Sensitive analyses of relationships of regular laxative use with risk of incident depression
