# Supplementary material for: Relationship of Regular Laxative Use, Genetic Susceptibility of Depression, and Risk of Incident Depression in the General Population
Source: Depress Anxiety. 2024 Oct 23;2024:6863037. doi: 10.1155/2024/6863037 (PMC11918892; doi:10.1155/2024/6863037)
Supplement: Supporting Information — Figure S1. Flow chart of the study participants. Figure S2. Standardized differences of participant characteristics before and after matched according to propensity scores by laxative use. Table S1. Postmatched participant characteristics according to propensity scores by laxative use. Table S2. Sensitive analyses of relationships of regular laxative use with risk of incident depression [file 6863037.f1.docx]

**Relationship of regular laxative use, genetic susceptibility of depression and risk of incident depression in the general population**

502,461 participants enrolled in the UK Biobank.

459,363 participants were included.

Excluded n=43,098

Withdrawn, n=107;

Having or missing data on depression at baseline, n= 42,991

450,045 participants included for final analysis.

Excluded n=9,318

Missing data on laxatives at baseline, n=9,318

.

**Supplemental Figure 1. Flow chart of the study participants.**

**
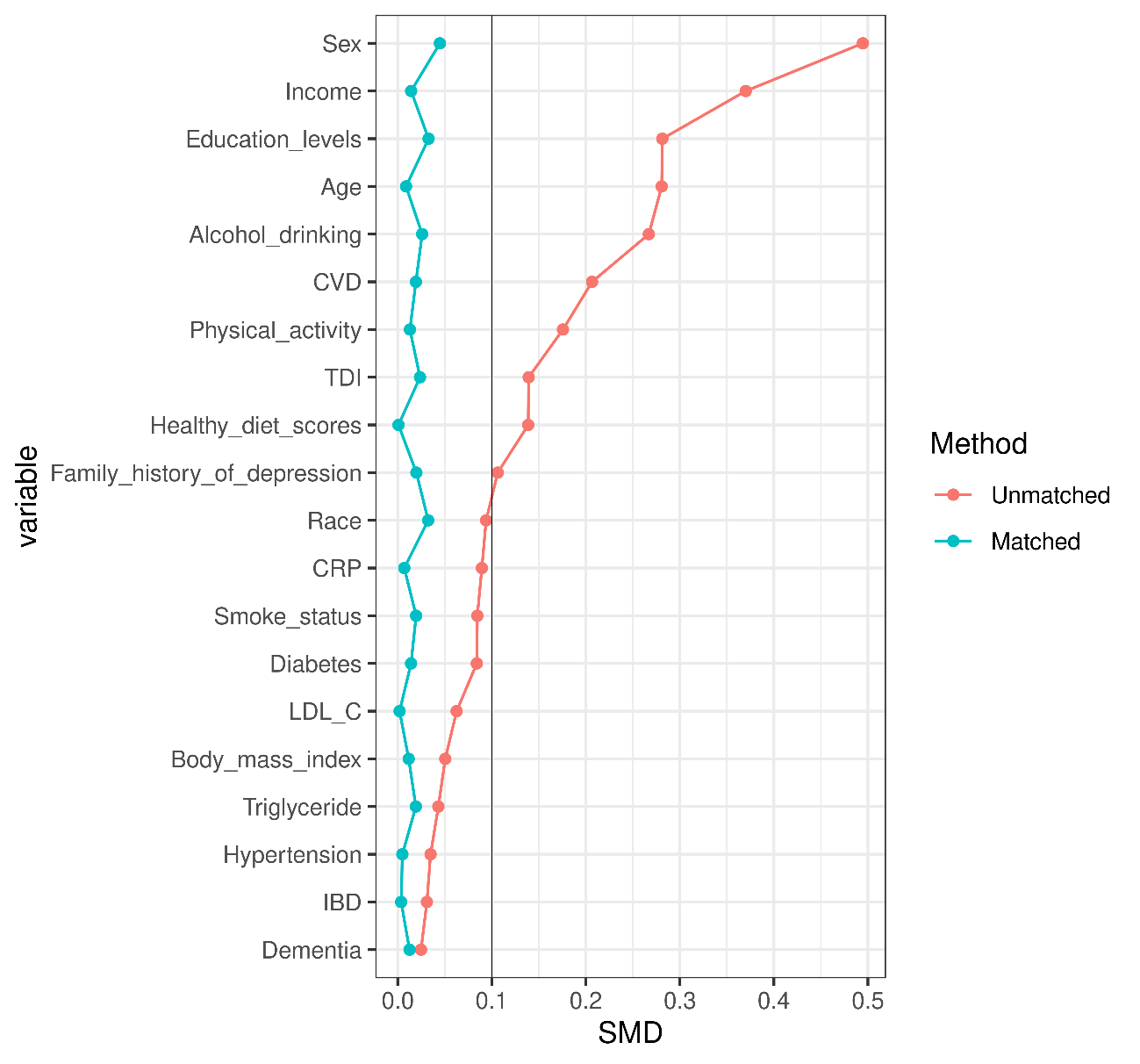
**

**Supplemental Figure 2. Standardized differences of participant characteristics before and after matched according to propensity scores by laxative use**

**Supplemental Table 1. Post-matched participant characteristics according to propensity scores by laxative use**

| **Baseline characteristics ^*^** | **Regular** **laxative use, n (%)** | | **Standardized**  **Difference, %** |
| --- | --- | --- | --- |
|  | **No** | **Yes** |  |
| N | 13,903 | 13,903 |  |
| Age, years | 58.7±7.6 | 58.7±7.6 | 0.9 |
| Male, n (%) | 3,131 (22.5) | 3,395 (24.4) | 4.5 |
| Body mass index, kg/m^2^ | 27.5±5.1 | 27.6±5.2 | 1.2 |
| White, n (%) | 12,967 (93.3) | 12,899 (92.8) | 3.2 |
| Townsend deprivation index | -1.04±3.2 | -0.96±3.3 | 2.4 |
| College or University degree, n (%) | 3,287 (23.6) | 3,247 (23.4) | 3.3 |
| Income less than 18,000£, n (%) | 3,837 (27.6) | 3,880 (27.9) | 1.4 |
| Family history of depression, n (%) | 2,017 (14.5) | 2,115 (15.2) | 2.0 |
| Healthy diet score | 3.2±1.4 | 3.2±1.4 | 0.1 |
| Smoke status, n (%) |  |  | 2.0 |
| Never | 7,205 (51.8) | 7,108 (51.1) |  |
| Ever | 5,249 (37.8) | 5,270 (37.9) |  |
| Current | 1,386 (10.0) | 1,460 (10.5) |  |
| Alcohol drinking, n (%) |  |  | 2.6 |
| Never | 1,636 (11.8) | 1,722 (12.4) |  |
| <1 time per week | 4,030 (29.0) | 4,020 (28.9) |  |
| 1-4 time per week | 5,940 (42.7) | 5,896 (42.4) |  |
| Daily or almost daily | 2,274 (16.4) | 2,251 (16.2) |  |
| Physical activity, n (%) |  |  | 1.3 |
| Low | 2,427 (17.5) | 2,490 (17.9) |  |
| Moderate | 4,320 (31.3) | 4,270 (30.7) |  |
| High | 3,901 (28.1) | 3,880 (27.9) |  |
| laboratory results |  |  |  |
| C-reactive protein, mg/L | 2.9±4.9 | 2.9±4.9 | 0.7 |
| LDL-C, mmol/L | 3.5±0.9 | 3.5±0.9 | 0.2 |
| Triglyceride, mmol/L | 1.7±1.0 | 1.7±0.9 | 1.9 |
| History of disease, n (%) |  |  |  |
| Hypertension | 7,950 (57.2) | 7,982 (57.4) | 0.5 |
| Diabetes | 1,058 (7.6) | 1,103 (7.9) | 1.4 |
| Cardiovascular disease | 1,659 (11.9) | 1,747 (12.6) | 1.9 |
| Inflammatory bowel disease | 197 (1.4) | 203 (1.5) | 0.4 |
| Dementia | 9 (0.1) | 14 (0.1) | 1.3 |

**^*^** Continuous variables are presented as mean ± SDs, category variables are presented as n (%).

Abbreviations: LDL-C, low density lipoprotein-cholesterol

**Supplemental Table 2. Sensitive analyses of relationships of regular laxative use with risk of incident depression**

| **Sensitive analyses** | **Non-regular laxative use** | | **Regular laxative use** | | **Adjusted HR (95%CI)*** |
| --- | --- | --- | --- | --- | --- |
|  | **N** | **Events (%)** | **N** | **Events (%)** |  |
| **Sensitive analysis 1** | 1,3903 | 694 (5.0) | 13,903 | 1,207 (8.7) | 1.79 (1.63,1.96) |
| **Sensitive analysis 2** | 430,698 | 15,282 (3.5) | 14,858 | 1,148 (7.7) | 1.75 (1.64,1.87) |
| **Sensitive analysis 3** | 421,323 | 11,389 (2.7) | 14,232 | 842 (5.9) | 1.73 (1.60,1.86) |
| **Sensitive analysis 4** | 413,745 | 14,952 (3.6) | 12,338 | 901 (7.3) | 1.68 (1.56,1.80) |
| **Sensitive analysis 5** | 434,840 | 17,322 (4.0) | 15,205 | 1,329 (8.7) | 1.79 (1.69,1.90) |
| **Sensitive analysis 6** | 434,840 | 17,322 (4.0) | 15,205 | 1,329 (8.7) | 1.76 (1.66,1.87) |

*Adjusted for age, sex, race, body mass index, education levels, household incomes, Townsend deprivation index, smoking and alcohol consumption status, family history of depression, physical activity, healthy diet scores, levels of low-density lipoprotein-cholesterol, triglyceride and C-reactive protein, prevalence of hypertension, diabetes, cardiovascular disease, inflammatory bowel disease and dementia.

**Sensitive analysis 1:** propensity score matching analysis was performed to evaluate the association of regular laxative use with risk of incident depression.

**Sensitive analysis 2:** The analysis was limited in participants who were followed for more than 2 years.

**Sensitive analysis 3:** The analysis was limited in participants who were followed for more than 5 years.

**Sensitive analysis 4:** The analysis was limited in participants excluding who have constipation at baseline or during follow-up.

**Sensitive analysis 5:** The analysis was further adjusted for genetic risk of depression.

**Sensitive analysis 6:** The analysis was further adjusted for history of abdominal operation.
